# Evolutionary Analysis Reveals a Single Amino Acid in the AAV Entry Receptor (AAVR) of Cats That Disrupts Binding of a Major Phylogenetic Group of AAVs

**DOI:** 10.3390/v18070744

**Published:** 2026-07-04

**Authors:** Edward E. Large, Isaac Mensah, Godfred Kumi, Michael S. Chapman

**Affiliations:** Department of Biochemistry, University of Missouri, Columbia, MO 65201, USA; largee@missouri.edu (E.E.L.); imd5w@missouri.edu (I.M.); gskngr@missouri.edu (G.K.)

**Keywords:** AAV5, AAVGo.1, AAV2, AAAV, AAVR, cell entry, virus receptor

## Abstract

Adeno-associated virus (AAV) is a small ssDNA satellite virus that receives wide attention due to its popularity as a safe and effective gene therapy vector. The AAV cell entry receptor (AAVR) for most serotypes is a glycoprotein containing five polycystic kidney disease (PKD) domains with which AAV interacts. AAV serotypes can be classified into three groups: those that interact primarily with PKD1, those whose interactions with PKD2 are stronger, and AAV4-like serotypes whose transduction is AAVR-independent. A phylogenetic analysis of AAVR and paralog KIAA0319 revealed AAVR amino acid variability in the region of PKD1 that is bound by AAV. We hypothesized that the substitution, in all cat-like animals, of a glutamate at a site that is an arginine (R353) in human AAVR may interfere with the binding of clade H AAVs that interact exclusively with PKD1. Analysis of PKD1 mutations, including ELISA, shows that an R353E substitution of glutamate for arginine affects the binding of the clade H AAVs that interact primarily with PKD1.

## 1. Introduction

Adeno-associated viruses (AAVs) are small single-stranded DNA viruses repurposed into effective and safe gene therapy delivery vehicles [1]. Each AAV capsid consists of 60 monomers of VP1:VP2:VP3 in a 1:1:10 ratio. The icosahedral surface exhibits three types of symmetry: 5-fold axes pass through each of twelve narrow pores, 3-fold axes relate elevated peaks that come together and the 2-fold axes relate neighboring peak triplets on either side of a surface depression [2,3].

Primate AAV serotypes are a popular choice for gene therapy and the AAV2 serotype is the canonical recombinant AAV (rAAV) vector [2,3]. AAV coding regions consist of two major open reading frames (ORFs), Rep and Cap, flanked by inverted terminal repeats (ITRs). In rAAV vectors, both the Rep and Cap ORFs are replaced by gene therapy cargo, with only the ITRs retained. During vector production via triple transfection, Rep and Cap proteins are supplied in trans from separate plasmids [4]. The Cap ORF amino acid sequence is a key determinant of tissue tropism and immune evasion properties [5,6,7].

**Figure 1:**
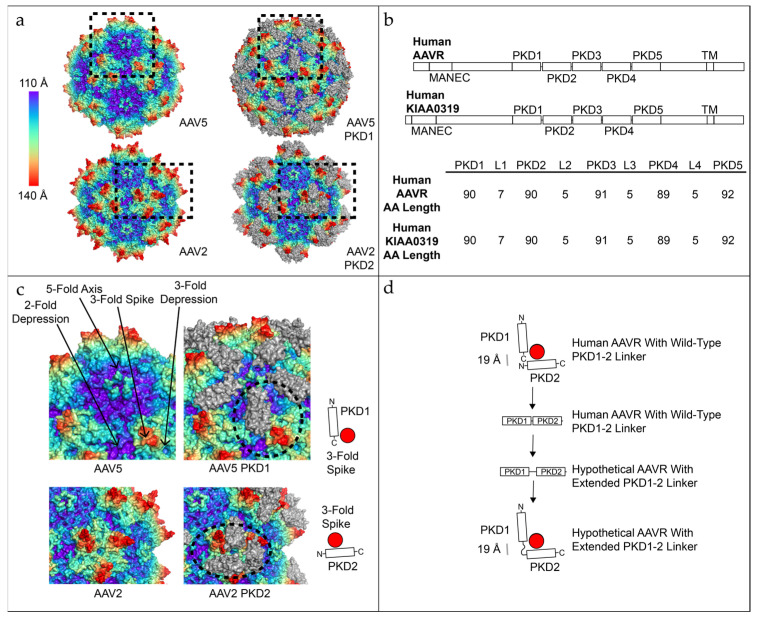
Two groups of AAVs bind to AAVR in distinct modes according to whether binding is exclusively to the PKD1 domain or stronger to the PKD2 domain. AAV has icosahedral symmetry, so there are 60 equivalent receptor-binding sites over the spherical surface. (**a**) Top row—AAV5, alone (**left**) or in complex (**right**) with PKD1 (gray). Bottom row—AAV2, alone (**left**) or in complex (**right**) with PKD2 (gray). (**b**) The human AAVR and KIAA0319 domains are shown schematically with their sizes (amino acids) and linker lengths. (**c**) Enlarged view of dotted line boxes in panel (**a**). (**d**) Schematic that first illustrates that the 7-residue linker is too short to bridge between the PKD1 and PKD2 locations as found in the complexes with AAV5 and AAV2 [13], but a hypothetical longer linker could support dual-domain binding.

AAV2 was instrumental in the discovery and characterization of a human proteinaceous AAV entry receptor (AAVR) [8]. AAVR is a glycoprotein (N-linked/O-linked) and contains three major protein regions. The N-terminus contains a motif at N-terminus with eight cysteines (MANEC) domain, while the central extracellular portion contains five polycystic kidney disease (PKD) domains, numbered 1 to 5 from N-terminus to C-terminus (Figure 1b). The C-terminus has a predicted transmembrane protein domain and a cytosolic domain responsible for trafficking AAV through the trans-Golgi network (TGN) [8]. AAVR has a paralog, KIAA0319, that has similar domains but is unable to bind AAV2. Domain swapping experiments demonstrate that human AAVR loses virus-binding activity when specific PKD domains are swapped between AAVR and KIAA0319 [9].

Structures of AAV:AAVR complexes for several serotypes have been determined [10,11,12,13,14,15]. AAVs can be classed into three groups according to the modes of AAVR binding: clade H (including AAV5 and AAVGo.1) binds exclusively to PKD1; most other clades (including AAV2 and AAV9) interact primarily with PKD2, but also PKD1, while clade G (AAV4-like serotypes) is AAVR-independent, using CD164 as a receptor [16,17]. Structures of clade H complexes show the AAVR PKD1 N-terminus above the virus near the 5-fold pore and the other end of the domain making contact with several AAV subunits near a viral 2-fold axis [11,13,14]. While many other AAVs are known to interact with both PKD2 and PKD1 domains [9], it is only PKD2 that has been reported as bound to the viral surface and well resolved in high-resolution structures of their complexes. PKD2 is bound with its N-terminus near the viral 2-fold axis, the domain’s ẞ-barrel rising up alongside the most prominent peak in the viral structure and its C-terminus extending outwards near the viral 3-fold, but not before the domain contacts four AAV subunits [11,12,15]. The PKD1 that was not resolved in the high-resolution structures has been visualized using lower resolution cryo-electron tomography (cryo-ET), pointing away from the viral surface [18]. A point to be emphasized is that the binding sites for PKD1 and PKD2 in their respective AAV groups involve distinct regions of the AAV surface (Figure 1a,c).

A conundrum is posed by the distinctiveness of the binding interactions: how did the AAV groups evolve to use discreetly different binding sites to interact with different domains of the same receptor? Was it possible that there existed either an extant or ancestral receptor with both domains bound by AAV? Could one imagine the two domains bound at different stages of entry or even simultaneously, absent a structure captured under conditions in which both domains of human AAVR are bound? Simplest would be to imagine adjacent PKD1 and PKD2 domains interacting with their respective sites as seen in the AAV5 and AAV2 structures. The linker region is not fully resolved in either structure, presumably due to disorder/flexibility. A total of 5 amino acids are not seen, but molecular modeling showed that 5 residues cannot bridge the inter-domain gap of 17 Å without unreasonable distortions to the domain structure [13]. The current study started with the question of whether, through phylogenetic analysis, a plausible inference could be made for an ancestral receptor with a sufficiently long linker sequence to enable simultaneous binding of both domains as seen in each of the prior structures (Figure 1d).

Below, we find that an ancestral form of AAVR (KIAA0319L) diverged from the paralog KIAA0319, following ancient gene duplication around the same time that the first AAVs arose in the paleovirological record. Thus, AAVR and KIAA0319 from a wide diversity of species were used for multiple sequence alignment and phylogenetic analysis. Variability between species was examined for multiple placental mammals (the best characterized hosts).

The sequence analysis highlighted a site of sequence variation within cats, the AAVR PKD1 domain at the equivalent residue to human R353, that is predicted by homology to be at the AAV-binding interface. The R353E variant is present in the common house cat and all cat-like animals. By homology to the structures of huAAVR complexed with AAV5 and AAVGo.1, R353 is expected to be a key contributor to the interface with AAV. Mutants were constructed to test the effects of mutations at this PKD1 site in a human and domestic house cat AAVR background using ELISA measures of binding.

## 2. Materials and Methods

### 2.1. AAV Production and Purification

The AAV5 virus-like particles (VLPs), consisting of empty capsid shells, were produced in Sf9 insect cells using the Bac-to-Bac baculovirus expression system with the pFastBacLIC plasmid (Addgene, Watertown, MA, USA, #30111). Proper expression of the VP1, VP2, and VP3 capsid proteins was achieved by incorporating the same translational control mutations described by [19] for AAV2 expression. The overall expression strategy followed previously established protocols for AAV2 [20], with the primary modification being the use of the pFastBacLIC plasmid as the bacmid shuttle. Because AAV5 exhibits weak affinity for heparin, the heparin affinity chromatography step commonly used during AAV2 purification was omitted. Instead, particle purification was completed using five rounds of cesium chloride density gradient ultracentrifugation, replacing the affinity chromatography step with two additional ultracentrifugation cycles.

### 2.2. PKD1 ELISAs

Two human and two cat PKD1 plasmids were ordered from Genscript. With the exception of one amino acid change, the two human and two cat plasmids are identical within species. Each plasmid consists of an N-terminus His-tag and stops before the linker region linking PKD1 to PKD2. Each plasmid was produced and purified from *E. coli* as previously described [20] in Rosetta cells. Briefly, a single transformed colony from a fresh LB–kanamycin (50 µg/mL) was plated into LB–kanamycin media at 5% of the final culture volume (e.g., 50 mL starter culture for a 1 L final culture). The culture was grown overnight at 37 °C with shaking at 210 rpm. The next day, the overnight culture was transferred into the final media volume and continued to incubate at 37 °C with shaking (210 rpm) until the OD600 reached ~1.0. 1 mL was removed as a pre-induction sample, pelleted at 6000× *g* for 1 min and stored at −20 °C for later SDS-PAGE analysis. IPTG (GoldBio, Olivette, MO, USA, cat.no. I2481) was added to the culture to a final concentration of 1 mM and incubated for 4–5 h at 37 °C with shaking (210 rpm). A 1 mL post-induction sample was collected and pelleted as above and stored at −20 °C. The remaining culture was pelleted at 6000× *g* and the supernatant was discarded. The cell pellet was lysed using a French press (Glen Mills, Inc., Clifton, NJ, USA) and the PKD1 proteins were purified using an IMAC column on an ÄKTA system (Cytiva, Malborough, MA, USA). Peak fractions were identified by SDS-PAGE and the desired fractions were pooled. The pooled protein was concentrated to approximately 1 mL using a 3 kDa MWCO Amicon Ultra centrifugal filter unit (MilliporeSigma, Burlington, MA, USA) for concentration determination and subsequent ELISA experiments.

The ELISA assay was performed as previously described [13], with minor modifications. Briefly, AAV5 capsids were diluted to 3 µg/mL in 100 mM sodium bicarbonate buffer, pH 9.6, and 50 µL was added to each well of a 96-well plate (Costar, Corning Inc., Corning, NY, USA, cat. no. 9018). Plates were incubated overnight at room temperature. Wells were then washed three times with TTBS (0.05% Tween-20, 50 mM Tris-HCl, and 150 mM sodium chloride, pH 7.5). Wells were blocked with 150 µL of 3% bovine serum albumin in TTBS (BSA; Fisher Scientific, Hampton, NH, USA, cat. no. BP9703-100) for 1 h and washed three times with TTBS. Afterwards, two-fold serial dilutions of human and cat PKD1 ligands, starting at 8 µM, were added to the wells, and incubated for 1 h. After three TTBS washes, 100 µL of anti-His antibody (Fisher Scientific, Hampton, NH, USA, cat. no. MA1-21315-HRP; 1:500 dilution) was added to each well and incubated for 1 h. Wells were washed three times with TTBS, followed by the addition of 90 µL TMB ELISA substrate (Abcam, Cambridge, UK, cat. no. ab171523). The reaction was developed for 2–5 min and stopped with 80 µL of 1 M hydrochloric acid. Absorbance was measured at 450 nm using a Synergy H1 plate reader (BioTek, Winooski, VT, USA). All incubation steps were performed at room temperature on a rocking platform.

### 2.3. Nonlinear Binding Analysis and Summary Metrics for ELISA Data

For each construct (hPKD1_wt, hPKD1_R353E, catPKD1_wt, catPKD1_E353R) and each independent experiment, ELISA response values were modeled using a one-site specific binding equation:

$$y=\frac{B_{max}\cdot x}{K_{d}+x},$$

where *x* is the ligand concentration and *y* is the measured ELISA signal. Curve fitting was performed using nonlinear least-squares regression implemented in the nlsLM algorithm within the minpack.lm package in R [21].

The following parameters were extracted:

- $K_{d}$(µM): concentration at half-maximal binding
- $B_{max}$(OD/RFU): fitted maximal ELISA response
- Initial slope: calculated as $B_{max}/K_{d}$, representing the model-predicted low-dose slope

Approximate 95% confidence intervals for *K_d_* and *B*_max_ were obtained using Wald-type intervals (estimate ± 1.96 × SE) derived from the fitted parameter covariance matrix.

To compare binding behavior between constructs, predefined construct pairs were evaluated within each independent experiment. These pairs included:

- hPKD1_wt vs. hPKD1_R353E
- catPKD1_wt vs. catPKD1_E353R
- catPKD1_E353R vs. hPKD1_wt
- catPKD1_wt vs. hPKD1_R353E

For each pair, fold-difference metrics were computed directly from the fitted parameters:

- Fold-*K_d_* = $K_{d,B}/K_{d,A}$
- Fold-*B*_max_ = $B_{max,B}/B_{max,A}$
- Fold-Slope = ${\left(B_{max}/K_{d}{)}_{B}/(B_{max}/K_{d}\right)}_{A}$

These values quantify relative affinity (*K_d_*), signal amplitude (*B*_max_), and initial steepness (slope) across constructs in each experiment. Calculations were performed only when both fits produced finite, positive estimates (Supplementary Table S1).

### 2.4. Expression Plasmid Construction and Sequences

Sequences were subcloned into the *E. coli* expression plasmid pET30a using Nde I and Hind III sites by Genscript. Both R353E and E353R are in bold and underlined:

hPKD1_wt
MASHHHHHHVSAGESVQITLPKNEVQLNAYVLQEPPKGETYTYDWQLITHP**R**DYSGEMEGKHSQILKLSKLTPGLYEFKVIVEGQNAHGEGYVNVTVKPEPRK*
hPKD1_R353E
MASHHHHHHVSAGESVQITLPKNEVQLNAYVLQEPPKGETYTYDWQLITHP**E**DYSGEMEGKHSQILKLSKLTPGLYEFKVIVEGQNAHGEGYVNVTVKPEPRK*
catPKD1_wt
MASHHHHHHVSAGKSVQITLPKNEVQLNAFVLQEPLEGETYTYDWQLITHP**E**DYS-GEMEGKHSQILKLSKLTPGLYEFRVIVDGQNTHGEGYVNVTVKPEPRK*
catPKD1_E353R
MASHHHHHHVSAGKSVQITLPKNEVQLNAFVLQEPLEGETYTYDWQLITHP**R**DYSGEMEGKHSQILKLSKLTPGLYEFRVIVDGQNTHGEGYVNVTVKPEPRK*

### 2.5. AAVR and KIAA0319 Family Sequence Identification and Analysis

**Figure 2:**
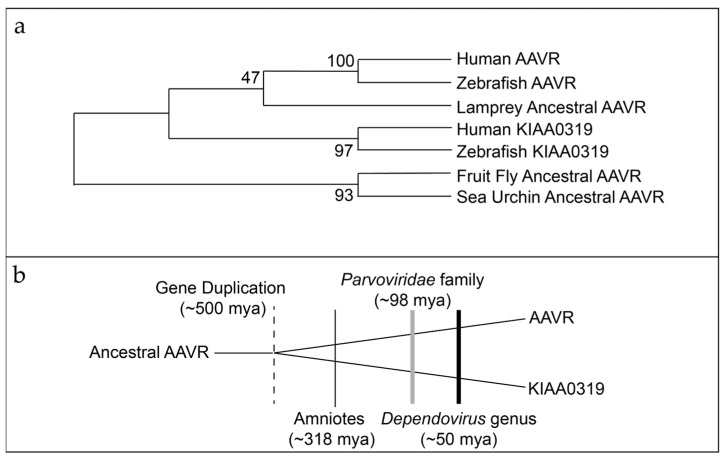
AAVR and KIAA0319 gene duplication. (**a**) Phylogenetic tree of AAVR/KIAA0319. Protostome (fruit fly) ancestral AAVR and basal deuterostome (sea urchin) ancestral AAVR form a group that does not have paralogs. KIAA0319 and AAVR are found in deuterostomes (human, zebrafish) with jawless vertebrates (lamprey) having only one AAVR. The numbers at each node show the bootstrap probability (500 replicates). (**b**) Duplication of the ancestral AAVR occurred before the emergence of amniotes (mammals, birds, and reptiles), parvoviruses and AAV. The dotted vertical line marks the duplication event in which the ancestral AAVR gene gave rise to AAVR and KIAA0319 approximately 500 million years ago. The thin gray vertical line indicates the origin of amniotes around 318 million years ago, the thick gray vertical line marks the emergence of the *Parvoviridae* family, and the thick black vertical line denotes the origin of the *Dependovirus* genus roughly 50 million years ago.

**Figure 3:**
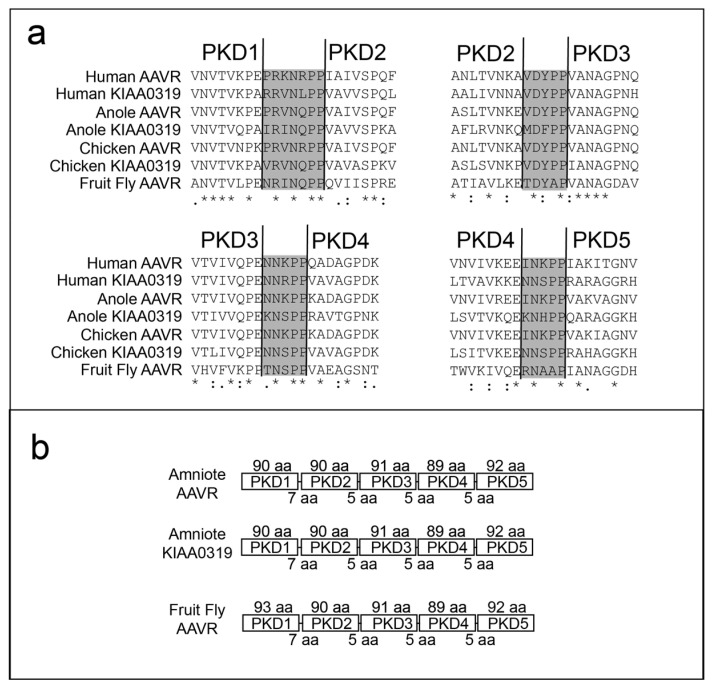
Conservation of the tandem repeat PKD domain structure within AAVR and KIAA0319. (**a**) Sequence alignment of the inter-domain linkers (gray) between amniotes and fruit fly, annotating lengths by numbers of amino acids (aa). Conserved amino acids are marked with “*”, conservative substitutions with “:”, and polar substitutions with “.”. (**b**) Domain sizes and linker lengths are highly conserved.

The NCBI sequence database was queried with human PKD15 AAVR domains iteratively through major metazoan, unicellular eukaryote, bacteria, archaea and plant protein sequence databases. No PKD hits were observed in plants, but PKD-like domains were detected with 20–30% sequence identity in unicellular eukaryotes, bacteria and archaea. Higher percent identity hits (>40%) were observed in metazoan genomes. The following sequences were used for Figure 1, Figure 2 and Figure 3: human AAVR (NP_079150), human KIAA0319 (NP_001161847), zebrafish AAVR (XP_068071433), zebrafish KIAA0319 (XP_001334630), lamprey AAVR (XP_032815244), fruit fly AAVR (NP_648171), sea urchin (XP_030830497), anole AAVR (XP_008116103), anole KIAA0319 (XP_008106997), chicken AAVR (XP_040545685), chicken KIAA0319 (XP_040519060), chimp AAVR (XP_009451884), rat AAVR (NP_001153127), mouse AAVR (NP_598647), guinea pig AAVR (XP_003471470), hamster AAVR (XP_007639789), rabbit AAVR (XP_008271916), domestic cat AAVR (XP_044891464), ferret AAVR (XP_004740871), dog AAVR (XP_038307976), pig AAVR (XP_020951570), and goat AAVR (XP_005678773).

For the AAVR phylogeny, the PKD15 sequences were examined using Jalview [22], aligned using Clustal O [23], and manually confirmed in MEGA7 [24]. Phylogenetic relationships were examined using maximum-likelihood in MEGA7 [24].

**Figure 4:**
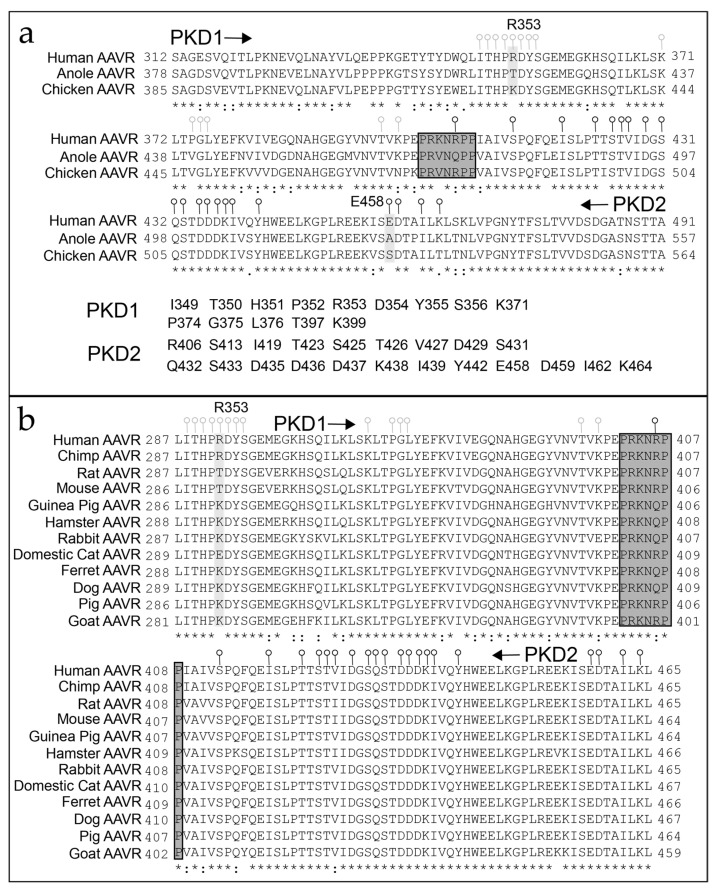
Conservation of AAV-binding regions of AAVR. (**a**) Amino acid variability between representative amniotes. PKD1 contact sites are shown with light gray circles and PKD2 contact sites are shown with black circles. The PKD1 contact sites were previously determined from a PKD1-2 complex with AAV2 at 2.4 Å [12] and AAV5 at 2.5 Å [13] using Coot [31], Relion [32] and RSRef [33]. The linker is highlighted in a dark gray box. (**b**) Amino acid sequence variation among representative placental mammals. Shown is the fragment of PKD1-2 sequence that contains all of the AAV contact amino acids shown in panel (**a**). Conserved amino acids are marked with “*”, conservative substitutions with “:”, and polar sub-stitutions with “.”.

Full alignments of proteins for Figure 2, Figure 3 and Figure 4 are shown in the Supplementary Information.

## 3. Results

### 3.1. AAVR/KIA00319L Domain Duplication and Divergence

Basal metazoans and jawless vertebrate genomes have a single ancestral AAVR-like gene, whereas both AAVR and KIAA0319 are found in jawed vertebrate lineages (Figure 2). Therefore, the duplication event between AAVR and KIAA0319 occurred sometime after the divergence of jawed vertebrates (gnathostomes) from jawless vertebrates (cyclostomes) ~500 million years ago (mya) [25]. Amniotes (mammals, birds and reptiles) differentiated approximately 330–350 mya [26]. The emergence of the *Parvoviridae* family and the *Dependoparvovirus* genus (i.e., AAVs) dates to an estimated 100 mya and 50 mya, respectively [27,28,29,30]. Therefore, AAVR and KIAA0319 were already well-established paralogs when AAV first entered the amniote lineage (Figure 2b).

### 3.2. PKD Linker Sequence Length Is Conserved in the AAVR/KIAA0319 Gene Family

The structures of complexes of several AAV serotypes with AAVR constructs have previously revealed which AAVR amino acids bind to AAV. It had been somewhat of a surprise that the strongest interactions of different serotypes were with different AAVR domains [9] and that the PKD1 and PKD2 domains were bound at mostly distinct sites on AAV [11,13]. In due course it would be realized that these represented two of three groups of AAV, the third accomplishing cell entry independently of AAVR [16,17]. Note that from high-resolution structures of AAVs complexed with multi-domain AAVR constructs, only single AAVR domains have been reported as immobilized on the viral surface, while lower resolution cryo-electron tomography (ET) has shown the neighboring domains pointing flexibly away from the viral surface [18]. Silveria et al. [13] wondered whether the diversity in AAVR binding sites could have evolved from an ancestral AAV that bound both domains of AAVR. However, they found that the linker between human PKD1 and PKD2 was too short to allow simultaneous binding of two domains of a single AAVR subunit if their binding poses were as found in the AAV5 and AAV2 complexes. As we considered the evolution of AAVR, an early question was whether there was any evidence of an ancestral AAVR with longer inter-domain linkers that would have allowed two-domain binding [13].

The evolutionary trajectory of AAVR and its paralog KIA00319 were examined. AAVR and KIAA0319 are conserved in animals and it was found that the PKD1-5 domains have constant spacing (Figure 3). The PKD1-2 linker is seven amino acids, of which four are fully conserved, including a proline pair. There is no evidence of longer linkers within any extant species that would be consistent with an ancestral AAVR subunit capable (as a monomer) of simultaneous two-domain binding. This does not preclude the possibility that there is (or has been during evolution) a temporal handoff between sites during entry.

### 3.3. AAVR Alleles with Potential Impact on AAV Binding in Animal Models

AAV vectors and the effects of transgene expression are often evaluated for potential clinical usage using non-human animal models. We assessed the distribution of sites showing divergent sequence variation in the AAVRs of placental mammals. We observed divergence at multiple amino acids that could potentially interfere with binding at the PKD1 and PKD2 sites (Figure 4). Of particular interest was a site (human AAVR R353) with significant variation (Figure 4). Earlier structural studies revealed R353 to be in intimate contact with the AAV capsid and it is also the approximate pivot point about which the PKD1 domain is rotated slightly in the complex of huAAVR with AAVGo.1 relative to AAV5 [13,14].

### 3.4. PKD12 Amino Acid Substitutions That May Distinguish AAV-Binding in the Cat Lineage

**Figure 5:**
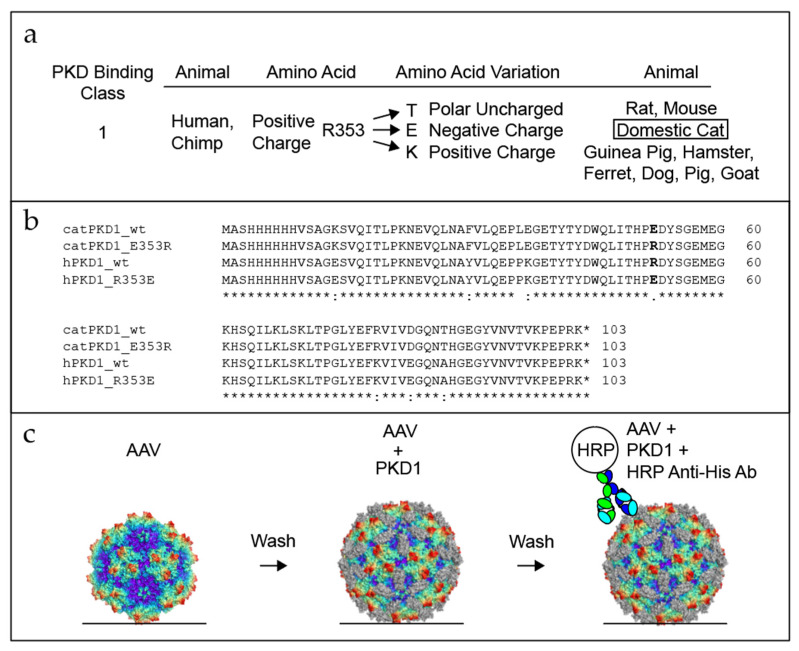
Development of an ELISA test to test PKD1 amino acid binding. (**a**) The human AAVR residue R353 within the PKD1 binding site is replaced by a variety of residue-types in different species (highlighted in bold). It can be a polar (but uncharged) threonine (T) or, more commonly, a positively charged arginine or lysine (R or K), but it is a negatively charged glutamate (E) in cats (wild and domestic). (**b**) Alignment of the PKD1 sequences used in this study, with human R353E and cat E353R shown in bold. (**c**) ELISA binding assay with AAV immobilized on the plate (horizontal line), then washed. PKD1 constructs are then incubated with the AAV, followed by an additional wash step. An antibody conjugated to horseradish peroxidase (HRP) is then added to determine binding affinity of PKD1. Conserved amino acids are marked with “*”, conservative substitutions with “:”, and polar substitutions with “.”.

The domestic cat variant R353E embodies an arginine to glutamic acid missense change that causes a conversion from a positive to a negatively charged amino acid (Figure 5). A survey of 42 broadly representative placental mammals indicates the R353E variant is found in 7 species (16.7%); 6 cat families (*Felidae*, tiger, lynx, cheetah, cougar, leopard, domestic cat) and one mongoose species (meerkat, family *Herpestidae*). Both the *Felidae* and *Herpestidae* families are part of the Carnivora order. The Carnivora order is divided into two suborders: the Feliformia (cat-like animals) and the Caniformia (dog-like animals). We observed R353E in Feliformia but not Caniformia species (3 bears, 2 seals, 1 ferret, 1 dog). The two Carnivora suborders diverged approximately 55 million years ago [34]; around the same time as the earliest evidence of the *Dependoparvovirus* genus. We hypothesized that R353E could have a substantial impact upon binding of clade H AAVs that interact primarily with PKD1 (like AAV5 or AAVGo.1). Experimental tests are reported later.

### 3.5. Evaluation of Domestic and Wild Cat Alleles Using ELISA

For characterization of the cat variant, we expressed human and domestic cat PKD1 with a His-tag at the N-terminus (Figure 5b: hPKD1_wt and catPKD1_wt). An R353E substitution was constructed in both hPKD1 and catPKD1 backgrounds (Figure 5b: hPKD1_R353E and catPKD1_E353R). Binding studies were by conventional ELISA.

**Figure 6:**
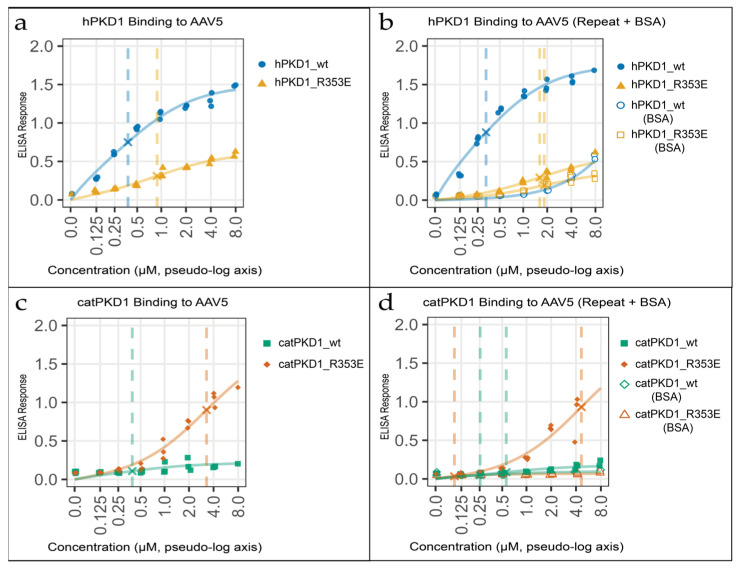
Residue 353 is a key determinant of AAV5-PKD1 binding. (**a**–**d**) AAV5 is immobilized on the plate for an indirect ELISA and probed with the addition of hPKD1_wt, hPKD1_R353E, catPKD1_wt or catPKD1_E353R. Panels: (**a**,**b**) hPKD1 repeat experiments, (**b**) including a negative control (BSA substituted for AAV5; hollow icons); (**c**,**d**) catPKD1 repeated experiments, (**d**) including a similar negative control. Curves are single site binding models fit to the data; dashed vertical lines denote K_d_; and “×” marks the half-max point (B_max_/2). A pseudo-log x-axis is used to declutter values near zero. Abbreviations: wt, wild type; hPKD1, human PKD1; catPKD1, domestic cat PKD1; BSA, bovine serum albumin.

We assayed a representative of the PKD1-binding group of AAVs, AAV5, and found dramatic differences. ELISA assays demonstrate that a glutamate at residue 353 in both human and domestic cat PKD1 significantly reduces AAV5 binding relative to an arginine at this position, indicating that residue 353 plays a critical role in mediating AAV5–PKD1 interactions. Across replicate experiments, hPKD1_R353E shows reduced binding affinity and a markedly diminished ELISA response, exhibiting a lower maximal signal and a much shallower dose–response relative to hPKD1_wt, whereas catPKD1_E353R demonstrates substantially reduced binding affinity but reaches a greatly elevated maximal ELISA signal compared with catPKD1_wt (Figure 6a–d; Supplementary Table S1). This result indicates the R353E variant severely impacts the binding of clade H AAVs. Therefore, domestic cats, and possibly the entire Carnivora suborder of Feliformia, have a single amino acid substitution that is expected to have a substantial impact upon the cell entry of clade H AAVs.

## 4. Discussion

In this study we evaluated the evolution of AAVR between species. We found that the duplication and divergence of AAVR and its paralog KIAA0319 occurred hundreds of millions of years before AAV. We also found an AAVR amino acid variant between species that falls within the PKD1 domain, and specifically within a part that forms the contact surface with the clade H AAV serotype, as seen in the structures of complexes of AAV with AAVR. Our experiments demonstrate a single variable amino acid site in domestic cats significantly affects class PKD1 binding. These observations provide valuable information for AAV researchers involved in the design and implementation of more effective AAV gene therapy delivery vectors.

We examined the evolution of AAVR and found that it is an ancient gene that diverged in the deuterostome lineage before AAV. An alternative possibility, now ruled out, had been that ancient AAVR duplicated after ancient AAV arose in the paleovirological lineage, which would have then required subsequent gain of AAV-binding susceptibility in AAVR or loss from KIAA0319. Instead, the ancient AAVR duplication event occurred ~500 mya and AAVs arose ~50 mya (Figure 2b). We also find that the linker length between PKD1 and PKD2 is conserved in both AAVR and KIAA0319, indicating a strict length requirement at this site that prevents AAV binding of AAVR PKD1 and PKD2 simultaneously. AAV, therefore, had at least 50 million years of evolutionary time to diverge into two different AAV groups that bind two distinct domains of AAVR (PKD1 and PKD2).

Finally, we describe a single variant in the common house cat that affects the ability of AAVR to interact with clade H (PKD1-binding) AAVs. Domestic cats are important models for human diseases such as cancer, diabetes, AIDS, and Alzheimer’s [35], so it is important to understand the susceptibility to different gene therapy vectors. It is also important for the development of non-surgical sterilization to control feral cat populations. Recent progress towards this goal has been achieved using AAV9, a PKD2-group binder, as a delivery system [36]. Perhaps it is fortunate that a PKD2 binding serotype was used for delivery instead of a clade H AAV.

Our results suggest that AAV5, a PKD1-group binder, would not be a good vector for experiments with domestic cat models. Indeed, in one cat brain study, AAV5-mediated transduction was on par with negative controls compared to AAV2 and AAV4, as evaluated by histochemistry and in situ hybridization [37]. A cat cochlea infection study with AAV5 elicited lower patterns of GFP transduction than mouse [38,39]. A panel of nine naturally occurring AAVs were tested for the infection potential of a broad range of feline cell lines and four out of nine AAVs, including AAV5, were ineffective based on EGFP expression [40]. However, in a cat retinal detachment study, AAV5 was used to express an inhibitor of apoptosis with moderate success [41]. In summary, apart from one study that appears to be an exception, AAV5 has proved empirically to be a poor vector in domestic cats and feline cell lines. Now there is a molecular rationale for these past empirical observations.

## Data Availability

The original contributions presented in this study are included in the article/Supplementary Materials. Further inquiries can be directed to the corresponding author(s).

## Supplementary Materials

The following supporting information can be downloaded at: https://www.mdpi.com/article/10.3390/v18070744/s1, Figure S1: Full alignment of AAVR and KIAAA0319 proteins described in Figure 2; Figure S2: Full alignment of AAVR and KIAAA0319 proteins described in Figure 3; Figure S3: Full alignment of AAVR proteins described in Figure 4; Table S1: ELISA-derived binding data for AAV-receptor complexes.

## Abbreviations

The following abbreviations are used in this manuscript:

AAV	Adeno-associated virus
AAVR	AAV cell entry receptor
VP	Viral protein
ORF	Open reading frame
TGN	Trans-Golgi network
MANEC	Motif at N-terminus with eight cysteines
PKD	Polycystic kidney disease
ET	Electron tomography
RSRef	Real-space atomic refinement
ELISA	Enzyme-linked immunosorbent assay
HRP	Horseradish peroxidase
