# Supplementary material for: Evolutionary Analysis Reveals a Single Amino Acid in the AAV Entry Receptor (AAVR) of Cats That Disrupts Binding of a Major Phylogenetic Group of AAVs
Source: Viruses. 2026 Jul 4;18(7):744. doi: 10.3390/v18070744 (PMC13431526; doi:10.3390/v18070744)
Supplement: Supplementary file 1 [file viruses-18-00744-s001.zip › viruses-3488663-supplementary.pdf]

Supplementary Information

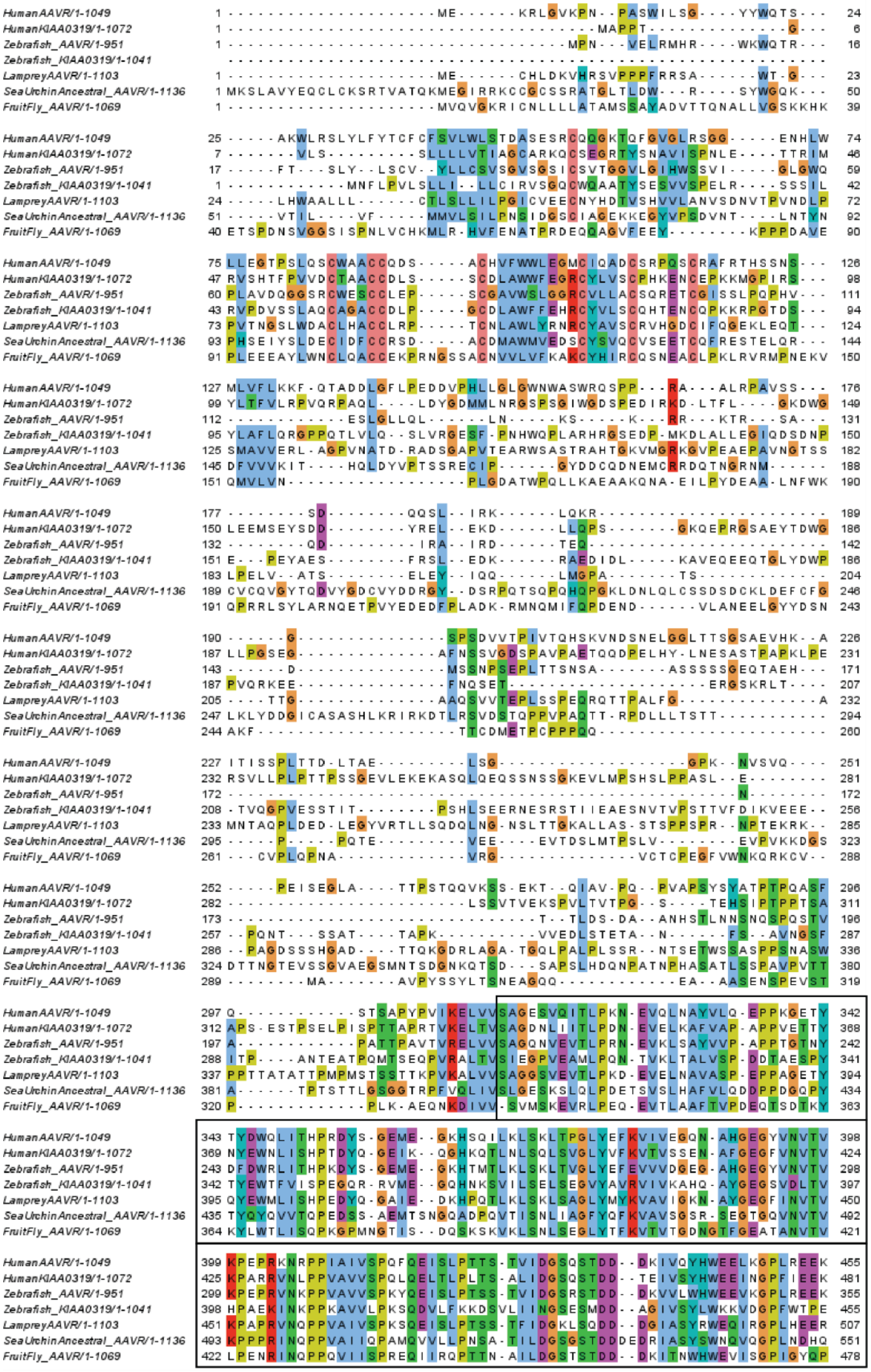

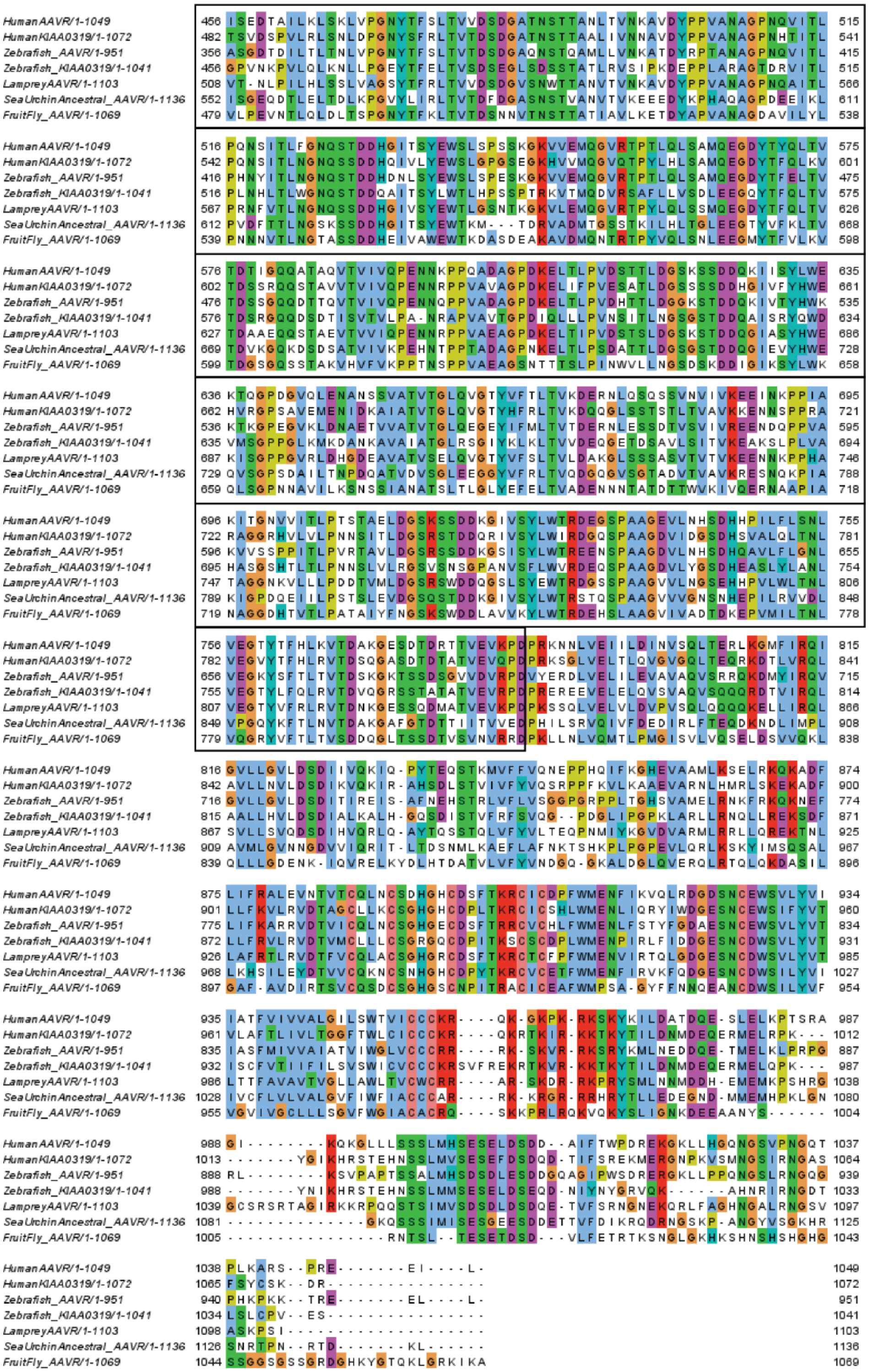

Supplementary Figure S1. Full alignment of AAVR and KIAAA0319 proteins described in Figure 2. Boxed alignments are PKD1-5. Sequences are colored using the Clustal scheme in Jalview.

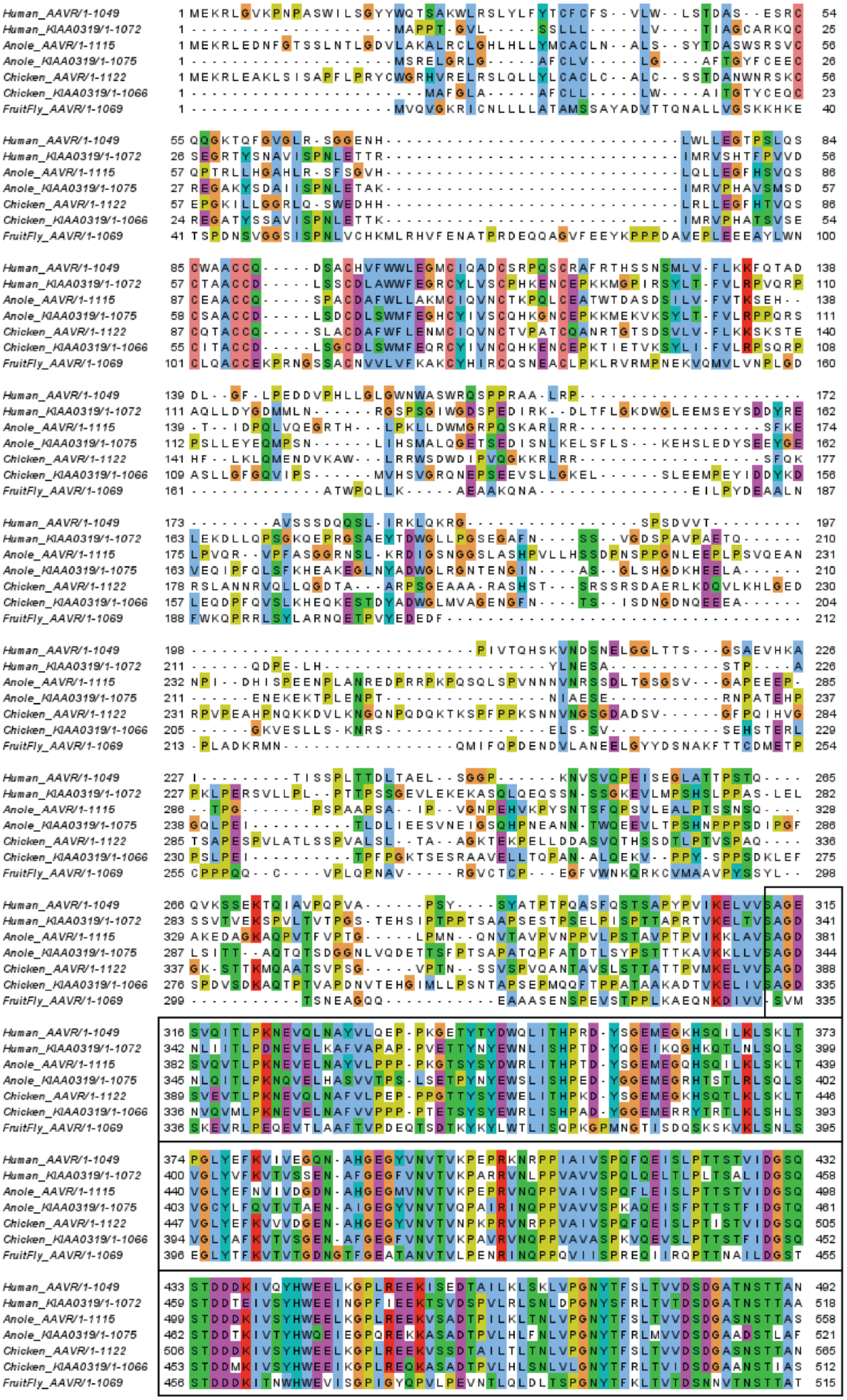
Human_AAVR/1-1049
Human_KIAA0319/1-1072
Anole_AAVR/1-1115
Anole_KIAA0319/1-1075
Chicken_AAVR/1-1122
Chicken_KIAA0319/1-1066
FruitFly_AAVR/1-1069

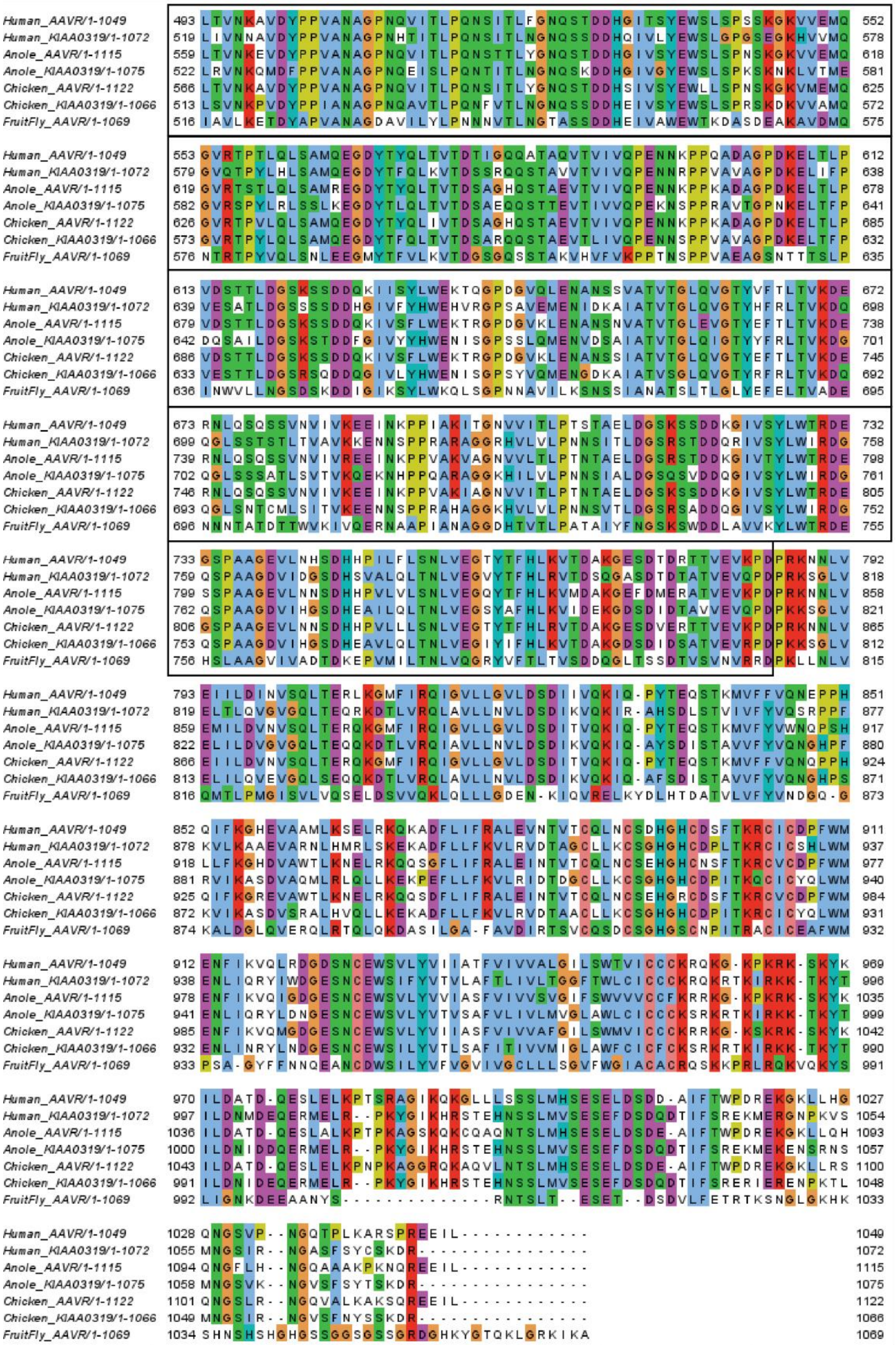

Supplementary Figure S2. Full alignment of AAVR and KIAAA0319 proteins described in Figure 3. Boxed alignments are PKD1-5. Sequences are colored using the Clustal scheme in Jalview.

```
Human_AAVR/1-1049            1 MEKRLGVKPNPASWILSGYYWQTSAKWLRSLYLFYTCFCFSVLWLSTDASESRCQQGKTQ  60
Chimp_AAVR/1-1049            1 MEKRLGVKPNPASWILSGYYWQTSVKWLRSLYLFYTCFCFSVLWLSTDASESRCQQGKTQ  60
Rat_AAVR/1-1049              1 MEKRLGVKPSTTSWVLPGYCWQISVKPPRSLYLVYSFFCLSVLWLSTDANESRCHQGKTL  60
Mouse_AAVR/1-1048            1 MEKRLGVKPSPASWVLPGYCWQTSVKLPRSLYLLYSFFCFSVLWLSTDADESRCQQGKTL  60
GuineaPig_AAVR/1-1048        1 MEKRLGVKPSPASWILSGYCWQTSVTCLRSLYLFYTCFCFGALWLSTDANESRCQQGKTQ  60
Hamster_AAVR/1-1050          1 MEKRLGVKPSPASWILPGYCWQTSVKPLRSLYLLYTFFCFSSLWLSTDANESRCHQGKTL  60
Rabbit_AAVR/1-1049           1 MEKRLGVMPVPASWILSGYCWQSTVKWLRSLYLCYACFCFSALWLSTDASESQCQQRKTQ  60
DomesticCat_AAVR/1-1086      1 MEKRLGVKPSPASWILSGFCWQLSVKWLRSLYLFFTCFCFSALWLSTDASESRCQHEKTE  60
Ferret_AAVR/1-1050           1 MEKRLGVKPSPASWILLGFCWQISVKWLRSLYPFYTCFCFSALWLSTDASESRCQQAKTE  60
Dog_AAVR/1-1051              1 MEKRLGVKPSPASWILLGFCWQISVKWLRSLYLFYTCFCFSALWLSTDASESRCQQAKTE  60
Pig_AAVR/1-1048              1 -MEKLGMKPSSASWILSRLCWQTSVKWFRSLYLFCICFCFSALWLTIDAKESRCQQAKTQ  59
Goat_AAVR/1-1043             1 -MEKLGIKPSRTSWILSRFCWQTFVKQFKSLYLFYICFCFSALWLSTDAKESRCQWVKTQ  59

Human_AAVR/1-1049           61 FGVGLRSGGENHLWLLEGTPSLQSCWAACCQDSACHVFWWLEGMCIQADCSRPQSCRAFR  120
Chimp_AAVR/1-1049           61 FGVGLRSGGENHLWLLEGTPSLQSCWAACCQDSACHVFWWLEGMCIQADCSRPQSCRAFR  120
Rat_AAVR/1-1049             61 YGAGLKTEGENHLRLLPGSLSFQACWAACCQDPACHALWWLEGMCLQADCSKPQSCQPFR  120
Mouse_AAVR/1-1048           61 YGAGLRTEGENHLRLLAGSLPFHACRAACCRDSACHALWWLEGMCFQADCSKPQSCQPFR  120
GuineaPig_AAVR/1-1048       61 FGVGLRSGGENHLWLLKGTPSLQSCWAACCLDSACHALWWLEGMCIQANCSKPQSCQTFR  120
Hamster_AAVR/1-1050         61 YGVGLRTGGENHLWLLKGSLSFQACWAVCCQDSACHALWWLEGMCFQADCSKPQSCQPFR  120
Rabbit_AAVR/1-1049          61 FGAGLRPGGENPLRLLEGSLSFQSCWAACCQDSACHAFWWLEGMCIQADCSKSRSCQAFR  120
DomesticCat_AAVR/1-1086     61 FGVGLRSGGENHLRLLEGTPSLQLCWAACCQDSACHAFWWLEGMCIQADCSRPRSCQTFR  120
Ferret_AAVR/1-1050          61 FGVGLRSGGENHLQLLEGTSSLQLCWAACCQDPACHAFWWLEGMCIQADCSRPQSCHIFR  120
Dog_AAVR/1-1051             61 FGVGLRSGGENHLRLLEGTSSLQLCWAACCQDSSCHAFWWLEGMCIQADCSSPQSCRAFR  120
Pig_AAVR/1-1048             60 FGVGLRSRGKNHLRLLEETPSFQSCWAACCQDSACHAFWWLEGICLQADCSRPQSCQAFR  119
Goat_AAVR/1-1043            60 FGVGLRSRGENHLRLLEGTPSLQSCWAACCQDSACHAFWWLEGMCIQADCSRPQSCQAFR  119

Human_AAVR/1-1049          121 THSSNSMLVFLKKFQTADDLGFLPEDDVPHLLGLGWNWASWR-QSPPRAALRPAVSSSDQ  179
Chimp_AAVR/1-1049          121 THSSNSMLVFLKKFQTADDLGFLPEDDVPHLLGLGWNWASWR-QSPPRAALRPAVSSSDQ  179
Rat_AAVR/1-1049            121 TDSSHSVLIVFQKSQTAKDLGLLPEDGEPHL-RRGWGRTSWRRQSLPGAPLTLSIPSSDH  179
Mouse_AAVR/1-1048          121 TDSSNSMLIIFQKSQTTDDLGLLPEDDEPHLLRLGWGRTSWRRQSLLGAPLTLSVPSSHH  180
GuineaPig_AAVR/1-1048      121 TDSSNSMLAFLKKFQMADDLGFLPEDDVPHFLGLGWSRASWRRQSPPRAPLRT-VSSNDQ  179
Hamster_AAVR/1-1050        121 TDCSNSILIFFQKSQTANDLGLLPEDDEPHLLRLGWGRTSWRRQSLPRAPGTLAVSFNDH  180
Rabbit_AAVR/1-1049         121 TDSSNSVLVFLKKFQTEDDLGFPPEDDVPHLLGLGWSRASWRRQGPPRAPLRLAVSSNDQ  180
DomesticCat_AAVR/1-1086    121 TDSSNSMLVFFKKFQTADGLGYPPEEDEPHILGLGWSRVSWRRQNPPRAPLRPTVSSSDQ  180
Ferret_AAVR/1-1050         121 TDSSNSMLVFFKKFQTADDLGFPPEDDVPHILGLGWSRTSWRRQSASRAPLRPTVSSGGH  180
Dog_AAVR/1-1051            121 TDSSNSMLVFFKKFQTADDLGFPPEDDVPHILGLGWSRTSWRRQRLPRALLRPTVSSSDH  180
Pig_AAVR/1-1048            120 TDSSNSILVFLKKFQIADDLDILLEDDVPHILGLDWSRASQRRQSLPRAPLRPTVSSSDQ  179
Goat_AAVR/1-1043           120 TDSSNSMLVFFQKFQIEDDLDFLPEDDVPHILGLDWSRASWRRWSPPRTPLRPPLS-SDQ  178

Human_AAVR/1-1049          180 QSLIRKLQKRGSPSDV-VTPIVTQHSKVNDSNELGGLTTSGSAEVHKAITISSPLTTDLT  238
Chimp_AAVR/1-1049          180 QSLIRKLQKRGSPSEV-VTPIVTQHSKVNDSNELGGLTTSGSAEVHKAITISSPLTTDLT  238
Rat_AAVR/1-1049            180 QSLLRERQKRDHPSVVP-TRVVIQQAKANHSEEASAASPRASAEVRKTITVPSSFTSNHT  238
Mouse_AAVR/1-1048          181 QSLLRDRQKRDL-SVVP-THGAMQHSKVNHSEEAGALSP-TSAEVRKTITVAGSFTSNHT  237
GuineaPig_AAVR/1-1048      180 QLLIRKLEKRESSSEIVP-SVVAQQSKVNESKELGGLNTSGSAEVHMEVKSSSPLTTNLT  238
Hamster_AAVR/1-1050        181 QSLTRARQKRDRLSEVS-PPGAVLHSKLNRSEEEGAVSPSASGEVRKAVTVPSPLTTDHT  239
Rabbit_AAVR/1-1049         181 RNLIRKLQKRENPSEVI--PLMTQHSKVNDSKELPDLTASGSTEVHKAVTVSSPSTTVLT  238
DomesticCat_AAVR/1-1086    181 QSLIRKLRKRDSPSEEVATHTVTQHSEMNDSKEVGDLNTSGSAEVHKAIVISSPLTTVLT  240
Ferret_AAVR/1-1050         181 HSLTRKLWKRDSPSEEVTTHAVTQH-EMNDSKEVGRLNTSGSAEVHKALIISSPLTPVLA  239
Dog_AAVR/1-1051            181 QSLTRKLWKRESPSEEVTTHIVTQHSEMNDSKEVGYLNTSGSAEVHKAVIISSPLTTVLT  240
Pig_AAVR/1-1048            180 QSLIRKLCKRESPSEEVA--AVTQPSKVNASKEPGDLNTTGSAEVHKAVIISNPLTTDQT  237
Goat_AAVR/1-1043           179 WSLIRKLRKRENPSEEVA--AVTQPSKTN---KLGDVNSTGSAEVHSTDTIFNPLTTDLS  233

Human_AAVR/1-1049          239 AELSGGPKNVSVQPEISEGLATTPSTQQVKSSEKTQIAVPQPVAPSYSYATPTPQASFQS  298
Chimp_AAVR/1-1049          239 AELSGGPKNVSVQPEISEGLATTPSTQQVKSSEKTQIAVPQPVAPSYSYATPTPQASFQS  298
Rat_AAVR/1-1049            239 TQTPEWPKNVSIPTEPSGHFTLASSTQQVKSTEHSPTDTPLPVAPSYSSATPTAQTSSQS  298
Mouse_AAVR/1-1048          238 TQTPEWPKNVSIHPEPSEHSSPVSGTPQVKSTEHSPTDAPLPVAPSYSYATPTPQASSQS  297
GuineaPig_AAVR/1-1048      239 AESPGWPKNVSVQPETSEDLDTALSSQQGKNPGKTQTA-AVPLPMATSYATPAPQASFQS  297
Hamster_AAVR/1-1050        240 TQTSDWPKNVSVHSEPPELTSPTSSTQQVKSTEHSPTDLPHPVAPSYNYATPTPQASSQS  299
Rabbit_AAVR/1-1049         239 AEIPDGPKNLSVQPEIPADPGTTASTPQVKSPGTMQVATPLPVAPSYSYATLTPKASFQS  298
DomesticCat_AAVR/1-1086    241 AETPGWSKNVSVQPETSEDPGATPSTQHIKSPGRIQIVTPLPVAPSYSYATPTPQASFQS  300
Ferret_AAVR/1-1050         240 AEIPGWSKNVSVQPETSEDPGTTPSTQQVKSLGKIQISTPLPVAPSYSYATPNPQASFQS  299
Dog_AAVR/1-1051            241 AEIPGWSKNESAQPETSEYPGTTPSTQQVKSLEKIQNATLLPVAPSYSYTTPDPQASFQT  300
Pig_AAVR/1-1048            238 EKIAGWSKNVSVQPETSEDPGTTSSVQQVKSPGKMQVATPLPVAPSYSYATSTPQTSFQS  297
Goat_AAVR/1-1043           234 VKIPGWSD-VSVQREASEDPSATPSIQQAKSPEEVLIAHPLPVAPSYSYATPTPQASFQS  292
```

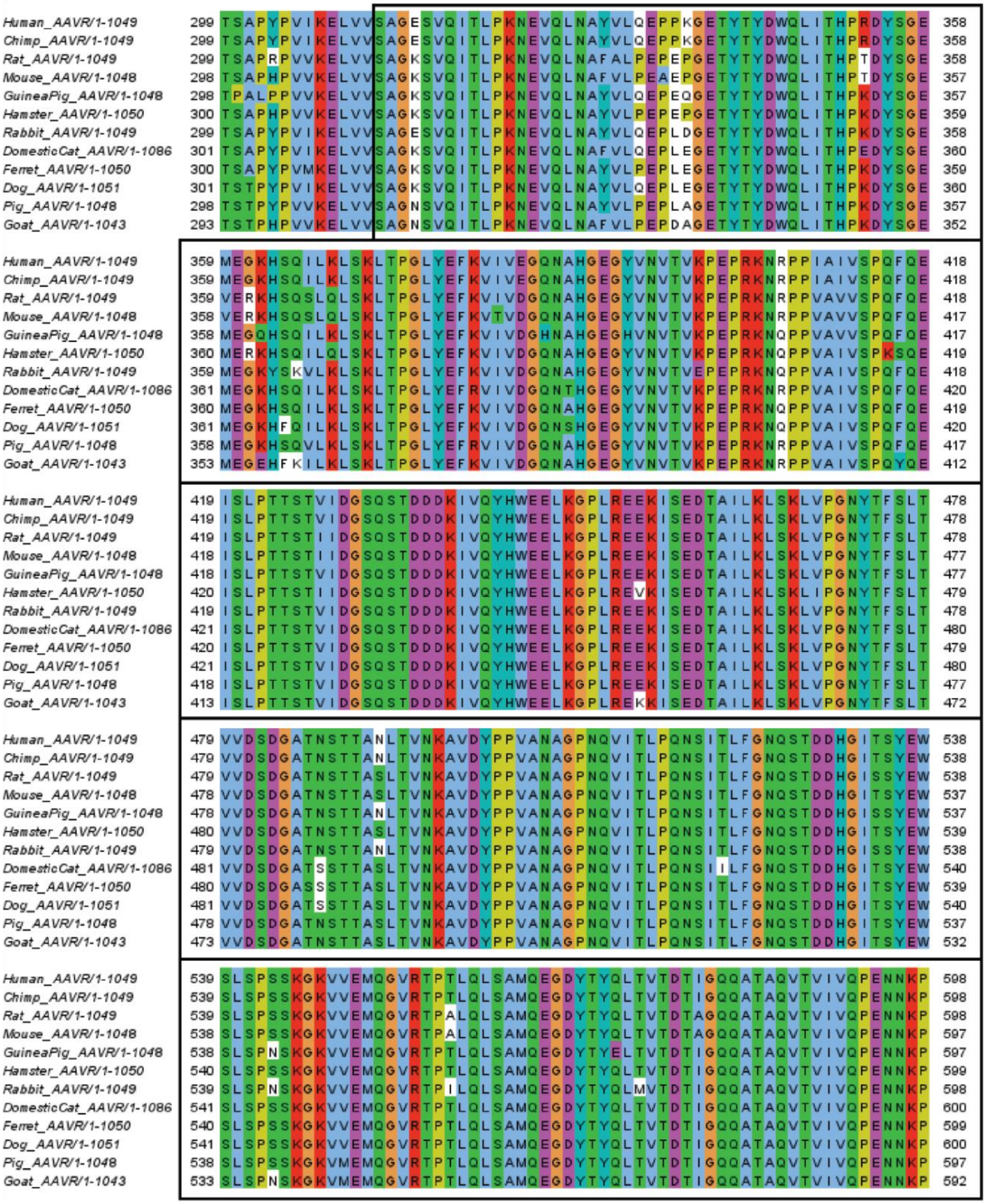
Human_AAVR/1-1049 299 TSAPYPVIKELVVSAGESVQITLPKNEVQLNAYVLQEPPKGETYTYDWQLITHPRDYSGE 358
Chimp_AAVR/1-1049 299 TSAPYPVIKELVVSAGESVQITLPKNEVQLNAYVLQEPPKGETYTYDWQLITHPRDYSGE 358
Rat_AAVR/1-1049 299 TSAPRPVVKELVVSAGKSVQITLPKNEVQLNAFALPEPEPGETYTYDWQLITHPTDYSGE 358
Mouse_AAVR/1-1048 298 TSAPHPVVKELVVSAGKSVQITLPKNEVQLNAFVLPEAEPGETYTYDWQLITHPTDYSGE 357
GuineaPig_AAVR/1-1048 298 TPALPPVVKELVVSAGKSVQITLPKNEVQLNAYVLQEPEQGETYTYDWQLITHPKDYSGE 357
Hamster_AAVR/1-1050 300 TSAPHPVVKELVVSAGKSVQITLPKNEVQLNAYVLPEPEPGETYTYDWQLITHPKDYSGE 359
Rabbit_AAVR/1-1049 299 TSAPYPVIKELVVSAGESVQITLPKNEVQLNAYVLQEPLDGETYTYDWQLITHPKDYSGE 358
DomesticCat_AAVR/1-1086 301 TSAPYPVIKELVVSAGKSVQITLPKNEVQLNAFVLQEPLEGETYTYDWQLITHPEDYSGE 360
Ferret_AAVR/1-1050 300 TSAPYPVMKELVVSAGKSVQITLPKNEVQLNAYVLPEPLEGETYTYDWQLITHPKDYSGE 359
Dog_AAVR/1-1051 301 TSTPYPVIKELVVSAGKSVQITLPKNEVQLNAYVLQEPLEGETYTYDWQLITHPKDYSGE 360
Pig_AAVR/1-1048 298 TSTPYPVVKELVVSAGNSVQITLPKNEVQLNAYVLPEPLAGETYTYDWQLITHPKDYSGE 357
Goat_AAVR/1-1043 293 TSTPHPVVKELVVSAGNSVQITLPKNEVQLNAFVLPEPDAGETYTYDWQLITHPKDYSGE 352
Human_AAVR/1-1049 359 MEGKHSQILKLSKLTPGLYEFKVIVEGQNAHGEGYVNVTVKPEPRKNRPPIAIVSPQFQE 418
Chimp_AAVR/1-1049 359 MEGKHSQILKLSKLTPGLYEFKVIVEGQNAHGEGYVNVTVKPEPRKNRPPIAIVSPQFQE 418
Rat_AAVR/1-1049 359 VERKHSQSLQLSKLTPGLYEFKVIVDGQNAHGEGYVNVTVKPEPRKNRPPVAVVSPQFQE 418
Mouse_AAVR/1-1048 358 VERKHSQSLQLSKLTPGLYEFKVTVDGQNAHGEGYVNVTVKPEPRKNRPPVAVVSPQFQE 417
GuineaPig_AAVR/1-1048 358 MEGQHSQILKLSKLTPGLYEFKVIVDGHNAHGEGHVNVTVKPEPRKNQPPVAVVSPQFQE 417
Hamster_AAVR/1-1050 360 MERKHSQILQLSKLTPGLYEFKVIVDGQNAHGEGYVNVTVKPEPRKNQPPVAIVSPKSQE 419
Rabbit_AAVR/1-1049 359 MEGKYSKVLKLSKLTPGLYEFKVIVDGQNAHGEGYVNVTVEPEPRKNQPPVAIVSPQFQE 418
DomesticCat_AAVR/1-1086 361 MEGKHSQILKLSKLTPGLYEFRVIVDGQNTHGEGYVNVTVKPEPRKNRPPVAIVSPQFQE 420
Ferret_AAVR/1-1050 360 MEGKHSQILKLSKLTPGLYEFKVIVDGQNAHGEGYVNVTVKPEPRKNQPPVAIVSPQFQE 419
Dog_AAVR/1-1051 361 MEGKHFQILKLSKLTPGLYEFKVIVDGQNSHGEGYVNVTVKPEPRKNQPPVAIVSPQFQE 420
Pig_AAVR/1-1048 358 MEGKHSQVLKLSKLTPGLYEFRVIVDGQNAHGEGYVNVTVKPEPRKNRPPVAIVSPQFQE 417
Goat_AAVR/1-1043 353 MEGEHFKILKLSKLTPGLYEFKVIVDGQNAHGEGYVNVTVKPEPRKNRPPVAIVSPQYQE 412
Human_AAVR/1-1049 419 ISLPTTSTVIDGSQSTDDDKIVQYHWEELKGPLREEKISEDTAILKLSKLVPGNYTFSLT 478
Chimp_AAVR/1-1049 419 ISLPTTSTVIDGSQSTDDDKIVQYHWEELKGPLREEKISEDTAILKLSKLVPGNYTFSLT 478
Rat_AAVR/1-1049 419 ISLPTTSTIIDGSQSTDDDKIVQYHWEELKGPLREEKISEDTAILKLSKLVPGNYTFSLT 478
Mouse_AAVR/1-1048 418 ISLPTTSTIIDGSQSTDDDKIVQYHWEELKGPLREEKISEDTAILKLSKLVPGNYTFSLT 477
GuineaPig_AAVR/1-1048 418 ISLPTTSTVIDGSQSTDDDKIVQYHWEELKGPLREEKISEDTAILKLSKLVPGNYTFSLT 477
Hamster_AAVR/1-1050 420 ISLPTTSTIIDGSQSTDDDKIVQYHWEELKGPLREVKISEDTAILKLSKLIPGNYTFSLT 479
Rabbit_AAVR/1-1049 419 ISLPTTSTVIDGSQSTDDDKIVQYHWEELKGPLREEKISEDTAILKLSKLVPGNYTFSLT 478
DomesticCat_AAVR/1-1086 421 ISLPTTSTVIDGSQSTDDDKIVQYHWEELKGPLREEKISEDTAILKLSKLVPGNYTFSLT 480
Ferret_AAVR/1-1050 420 ISLPTTSTVIDGSQSTDDDKIVQYHWEELKGPLREEKISEDTAILKLSKLVPGNYTFSLT 479
Dog_AAVR/1-1051 421 ISLPTTSTVIDGSQSTDDDKIVQYHWEELKGPLREEKISEDTAILKLSKLVPGNYTFSLT 480
Pig_AAVR/1-1048 418 ISLPTTSTVIDGSQSTDDDKIVQYHWEELKGPLREEKISEDTAILKLSKLVPGNYTFSLT 477
Goat_AAVR/1-1043 413 ISLPTTSTVIDGSQSTDDDKIVQYHWEELKGPLREKKISEDTAILKLSKLVPGNYTFSLT 472
Human_AAVR/1-1049 479 VVDSDGATNSTTANLTVNKAVDYPPVANAGPNQVITLPQNSITLFGNQSTDDHGITSYEW 538
Chimp_AAVR/1-1049 479 VVDSDGATNSTTANLTVNKAVDYPPVANAGPNQVITLPQNSITLFGNQSTDDHGITSYEW 538
Rat_AAVR/1-1049 479 VVDSDGATNSTTASLTVNKAVDYPPVANAGPNQVITLPQNSITLFGNQSTDDHGISSYEW 538
Mouse_AAVR/1-1048 478 VVDSDGATNSTTASLTVNKAVDYPPVANAGPNQVITLPQNSITLFGNQSTDDHGITSYEW 537
GuineaPig_AAVR/1-1048 478 VVDSDGATNSTTANLTVNKAVDYPPVANAGPNQVITLPQNSITLFGNQSTDDHGISSYEW 537
Hamster_AAVR/1-1050 480 VVDSDGATNSTTASLTVNKAVDYPPVANAGPNQVITLPQNSITLFGNQSTDDHGITSYEW 539
Rabbit_AAVR/1-1049 479 VVDSDGATNSTTANLTVNKAVDYPPVANAGPNQVITLPQNSITLFGNQSTDDHGISSYEW 538
DomesticCat_AAVR/1-1086 481 VVDSDGATSSTTASLTVNKAVDYPPVANAGPNQVITLPQNSIILFGNQSTDDHGITSYEW 540
Ferret_AAVR/1-1050 480 VVDSDGASSSTTASLTVNKAVDYPPVANAGPNQVITLPQNSITLFGNQSTDDHGITSYEW 539
Dog_AAVR/1-1051 481 VVDSDGATSSTTASLTVNKAVDYPPVANAGPNQVITLPQNSITLFGNQSTDDHGITSYEW 540
Pig_AAVR/1-1048 478 VVDSDGATNSTTASLTVNKAVDYPPVANAGPNQVITLPQNSITLFGNQSTDDHGITSYEW 537
Goat_AAVR/1-1043 473 VVDSDGATNSTTASLTVNKAVDYPPVANAGPNQVITLPQNSITLFGNQSTDDHGITSYEW 532
Human_AAVR/1-1049 539 SLSPSSKGKVVEMQGVRTPTLQLSAMQEGDYTYQLTVTDTIGQQATAQVTVIVQPENNKP 598
Chimp_AAVR/1-1049 539 SLSPSSKGKVVEMQGVRTPTLQLSAMQEGDYTYQLTVTDTIGQQATAQVTVIVQPENNKP 598
Rat_AAVR/1-1049 539 SLSPSSKGKVVEMQGVRTPALQLSAMQEGDYTYQLTVTDTAGQQATAQVTVIVQPENNKP 598
Mouse_AAVR/1-1048 538 SLSPSSKGKVVEMQGVRTPALQLSAMQEGDYTYQLTVTDTAGQQATAQVTVIVQPENNKP 597
GuineaPig_AAVR/1-1048 538 SLSPNSKGKVVEMQGVRTPTLQLSAMQEGDYTYELTVTDTIGQQATAQVTVIVQPENNKP 597
Hamster_AAVR/1-1050 540 SLSPSSKGKVVEMQGVRTPTLQLSAMQEGDYTYQLTVTDTIGQQATAQVTVIVQPENNKP 599
Rabbit_AAVR/1-1049 539 SLSPNSKGKVVEMQGVRTPILQLSAMQEGDYTYQLMVTDTIGQQATAQVTVIVQPENNKP 598
DomesticCat_AAVR/1-1086 541 SLSPSSKGKVVEMQGVRTPTLQLSAMQEGDYTYQLTVTDTIGQQATAQVTVIVQPENNKP 600
Ferret_AAVR/1-1050 540 SLSPSSKGKVVEMQGVRTPTLQLSAMQEGDYTYQLTVTDTIGQQATAQVTVIVQPENNKP 599
Dog_AAVR/1-1051 541 SLSPSSKGKVVEMQGVRTPTLQLSAMQEGDYTYQLTVTDTIGQQATAQVTVIVQPENNKP 600
Pig_AAVR/1-1048 538 SLSPSSKGKVMEMQGVRTPTLQLSAMQEGDYTYQLTVTDTIGQQATAQVTVIVQPENNKP 597
Goat_AAVR/1-1043 533 SLSPNSKGKVMEMQGVRTPTLQLSAMQEGDYTYQLTVTDTIGQQATAQVTVIVQPENNKP 592

```
Human_AAVR/1-1049          599 PQADAGPDKELTLPVDSTTLDGSKSSDDQKIISYLWEKTQGPDGVQLENANSSVATVTGL 658
Chimp_AAVR/1-1049          599 PQADAGPDKELTLPVDSTTLDGSKSSDDQKIISYLWEKTQGPDGVQLENANSSVATVTGL 658
Rat_AAVR/1-1049            599 PQADAGPDKELTLPVDSTTLDGSKSTDDQRVVSYLWEQSRGPDGVQLENANSSVATVTGL 658
Mouse_AAVR/1-1048          598 PQADAGPDKELTLPVDSTTLDGSKSTDDQRVVSYLWEQSRGPDGVQLENANSSVATVTGL 657
GuineaPig_AAVR/1-1048      598 PQADAGPDKELTLPVDSTTLDGSKSSDDQRIASYLWEKTQGPDGVQLENANSSVATVTGL 657
Hamster_AAVR/1-1050        600 PQADAGPDKELTLPVDSTSLDGSKSTDDQRIVSYVWEKTQGPDGVQLENANSSVATVTGL 659
Rabbit_AAVR/1-1049         599 PQADAGPDKELTLPVDSTTLDGSKSSDDQRIASYLWEKTQGPDGVQLENANSSVATVTGL 658
DomesticCat_AAVR/1-1086    601 PQADAGPDKELTLPVDSTTLDGSKSSDDQKIISYLWEKTQGPDGVQLENANSSVATVTGL 660
Ferret_AAVR/1-1050         600 PQADAGPDKELTLPVDSTTLDGSKSSDDQKIISYLWEKTQGPDGVQLENANSSVATVTGL 659
Dog_AAVR/1-1051            601 PQADAGPDKELTLPVDSTTLDGSKSSDDQKIISYLWEKTQGPDGVQLENANSSVATVTGL 660
Pig_AAVR/1-1048            598 PQADAGPDKELTLPVDSTTLDGSKSSDDQKIISYLWEKTQGPDGVQLENANSSVATVAGL 657
Goat_AAVR/1-1043           593 PQADAGPDKELTLPVDSTTLDGSKSSDDQKIISYLWEKTQGPDGVQLENANSSVATVTGL 652

Human_AAVR/1-1049          659 QVGTYVFTLTVKDERNLQSQSSVNVIVKEEINKPPIAKITGNVVITLPTSTAELDGSKSS 718
Chimp_AAVR/1-1049          659 QVGTYVFTLTVKDERNLQSQSSVNVIVKEEINKPPIAKITGNVVITLPTSTAELDGSKSS 718
Rat_AAVR/1-1049            659 QVGTYVFTLTVKDERNLQSQSSVSVIVKEEINKPPVAKITGNVVITLPTSTVELDGSRSS 718
Mouse_AAVR/1-1048          658 QVGTYVFTLTVKDERNLQSQSSVNVIVKEEINKPPVAKIAGNVVVTLPTSTAELDGSRSS 717
GuineaPig_AAVR/1-1048      658 QVGTYVFTLTVKDERNLQSQSSVNVIVKEEMNKPPVAKITGNVVIILPTNTAELDGSKSS 717
Hamster_AAVR/1-1050        660 QVGTYVFTLTVKDERNLQSRSSVNVIVKEEINKPPVAKITGNVVVTLPTSTAELDGSRSS 719
Rabbit_AAVR/1-1049         659 QVGTYVFTLTVKDERNLQSQSSVNVIVREEMNKPPTARITGNVVITLPINTAELDGSKSS 718
DomesticCat_AAVR/1-1086    661 QVGTYVFTLTVKDERNLQSQSSVNVIVKEEMNKPPVAKITGSVVITLPTDTAELDGSKSS 720
Ferret_AAVR/1-1050         660 QVGTYVFTLTVKDERNLQSQSSVNVIVKEEMNKPPVAKITGNVVITLPTDTAELDGSKSS 719
Dog_AAVR/1-1051            661 QVGTYVFTLTVKDERNLQSQSSVNVIVKEEMNKPPIAKITGNVVITLPTDTAELDGSKSS 720
Pig_AAVR/1-1048            658 QVGTYVFTLTVRDERNLQSQSSVNVIVKEEMNKPPIAKITGIVVITLPTDTAELDGSKSS 717
Goat_AAVR/1-1043           653 QVGTYVFTLTVRDERNLQSQSSVNVIVKEEMNKAPVAKIAGNVVITLPTDTAELDGSKSS 712

Human_AAVR/1-1049          719 DDKGIVSYLWTRDEGSPAAGEVLNHSDHHPILFLSNLVEGTYTFHLKVTDAKGESDTDRT 778
Chimp_AAVR/1-1049          719 DDKGIVSYLWTRDEGSPAAGEVLNHSDHHPILFLSNLVEGTYTFHLKVTDAKGESDTDRT 778
Rat_AAVR/1-1049            719 DDKGIVSYLWTRDEASPAAGEVLNHSDHHPVLFLSNLVEGSYTFHLKVTDAKGESDTDRT 778
Mouse_AAVR/1-1048          718 DDKGIVSYLWTRDETSPAAGEVLNHSDHHPVLFLSNLVEGTYTFHLKVTDAKGESDTDRT 777
GuineaPig_AAVR/1-1048      718 DDKGIINYVWTRDEGSPAAGEVLNHSDHHPILFLSNLVEGTYTFHLKVTDAKGESDIDRA 777
Hamster_AAVR/1-1050        720 DDKGIVSYLWTRDETSPAAGEVLNHSDHHPVLFLSNLVEGTYTFHLKVTDAKGESDTDRT 779
Rabbit_AAVR/1-1049         719 DDKGIVSYHWTRDEGSPAAGEVLNHSDHHPVLFLSNLVEGTYTFHLKVTDAKGESDTDRT 778
DomesticCat_AAVR/1-1086    721 DDKAIVSYLWTRDEGSPAAGEVLNHSDHHPILFLSNLVEGTYTFHLHVTDAKGESDTDRT 780
Ferret_AAVR/1-1050         720 DDKGIVSYLWTRDEGSPAAGEVLNHSDHHPILFLSNLVEGTYTFHLHVTDAKGESDMDRT 779
Dog_AAVR/1-1051            721 DDKGIVSYLWTRDEGSPAAGEVLNHSDHHPILFLSNLVEGTYTFHLHVTDAKGESDMDRT 780
Pig_AAVR/1-1048            718 DDKGIVSYLWTRDEGSPAAGDVLNHSDHHPILFLSNLVEGTYTFHLKVTDAKGESDTDRT 777
Goat_AAVR/1-1043           713 DDKGIVSYLWTRDEGSPAAGEVLNRSDHHPVLFLSNLVEGTYTFHLKVTDAKGESDVDRT 772

Human_AAVR/1-1049          779 TVEVKPDPRKNNLVEIILDINVSQLTERLKGMFIRQIGVLLGVLDSDIIVQKIQPYTEQS 838
Chimp_AAVR/1-1049          779 TVEVKPDPRKNNLVEIILDINVSQLTERLKGMFIRQIGVLLGVLDSDIIVQKIQPYTEQS 838
Rat_AAVR/1-1049            779 TVEVKPDPRKSNLVEIILDVNVSQLTERLKGMFIRQIGVLLGVLDSDIIVQKIQPYTEQS 838
Mouse_AAVR/1-1048          778 TVEVKPDPRKSNLVEIILDVNVSQLTERLKGMLIRQIGVLLGVLDSDIIVQKIQPYTEQS 837
GuineaPig_AAVR/1-1048      778 TVEVKPDPMKNNLVEIILDVSVSQLTERLKGMFIRQIGVLLGVLDSDIIVQKIQPYTEQS 837
Hamster_AAVR/1-1050        780 TVEVKPDPRKNNLVEIILDVNVSQLTERLKGMFIRQIGVLLGVLDSDIIVQKIQPYTEQS 839
Rabbit_AAVR/1-1049         779 TVEVKPDPRKNNLVEIILDVNVSQLTERLKGMFIRQIGVLLGVLDSDIIVQKIQPYTEQS 838
DomesticCat_AAVR/1-1086    781 TVEVKPDPRKNNLVEIILDVNVSQLTERLKGMFIRQIGVLLGVLDSDIIVQKIQPYTEQS 840
Ferret_AAVR/1-1050         780 TVEVKPDPRKNHLVEIILDVNVSQLTERLKGMFIRQIGVLLGVLDSDIIVQKIQPYTEQS 839
Dog_AAVR/1-1051            781 TVEVKPDPRKNNLVEIILDVNVSQLTERLKGMFIRQIGVLLGVLDSDIIVQKIQPYTEQS 840
Pig_AAVR/1-1048            778 TVEVKPDPRKNNLVEIILDVNVSQLTERLKGMFIRQIGVLLGVLDSDIIVQKIQPYTEQS 837
Goat_AAVR/1-1043           773 TVEVKPDPRKNSLVEIILDVNVSQLTERLKGMFIRQIGVLLGVLDSDIIVQKIQPYTEQS 832

Human_AAVR/1-1049          839 TKMVFFVQNEPPHQIFKGHEVAAMLKSELRKQKADFLIFRALEVNTVTCQLNCSDHGHCD 898
Chimp_AAVR/1-1049          839 TKMVFFVQNEPPHQIFKGHEVAAMLKSELRKQKADFLIFRALEVNTVTCQLNCSDHGHCD 898
Rat_AAVR/1-1049            839 TKMLFFVQNDPPHQFFKGREVAAMLKSELRKQKADFLIFRALEINTVTCQLNCSDHGHCD 898
Mouse_AAVR/1-1048          838 TKMLFFVQNDPPHQLFKGHEVAAMLKSELQKQKADFLIFRALEISTVTCQLNCSDHGHCD 897
GuineaPig_AAVR/1-1048      838 TKMVFYVQNEPPHQIFKGYEVAAMLKNELRKQKADFLIFGALEINTVTCQLNCSDHGHCD 897
Hamster_AAVR/1-1050        840 TKMLFFVQNEPPHQLFKGHEVAAMLKSELRKQKADFLIFRALEINTVTCQLNCSDHGHCD 899
Rabbit_AAVR/1-1049         839 TKMVFFVQNEPAHQIFKGHQVAAMLKSELRKQKADFLIFGALEISTVTCQLNCSDHGHCD 898
DomesticCat_AAVR/1-1086    841 TKMVFFVQNEPPHQIFKGHEVAAMLKNELRKQKADFLIFRALEINTVTCQLNCSDHGHCD 900
Ferret_AAVR/1-1050         840 TKMVFFVQNEPPHQIFKGHEVAAMLKSELRKQKADFLIFRALEINTVTCQLNCSDHGHCD 899
Dog_AAVR/1-1051            841 TKMVFFVQNEPPNQIFKGHEVAAMLKSELQKQKADFLIFRALEINTVTCQLNCSDHGHCD 900
Pig_AAVR/1-1048            838 TKMVFFVQNEPPHQIFKGHEVAAMLKSELQKQKADFLIFRALEINTVTCQLNCSDHGHCD 897
Goat_AAVR/1-1043           833 TKMVFFVQNEPPHQIFKGHEVAAMLKSELQKQKTDFLIFRALEINTVTCQLNCSDHGHCD 892
```

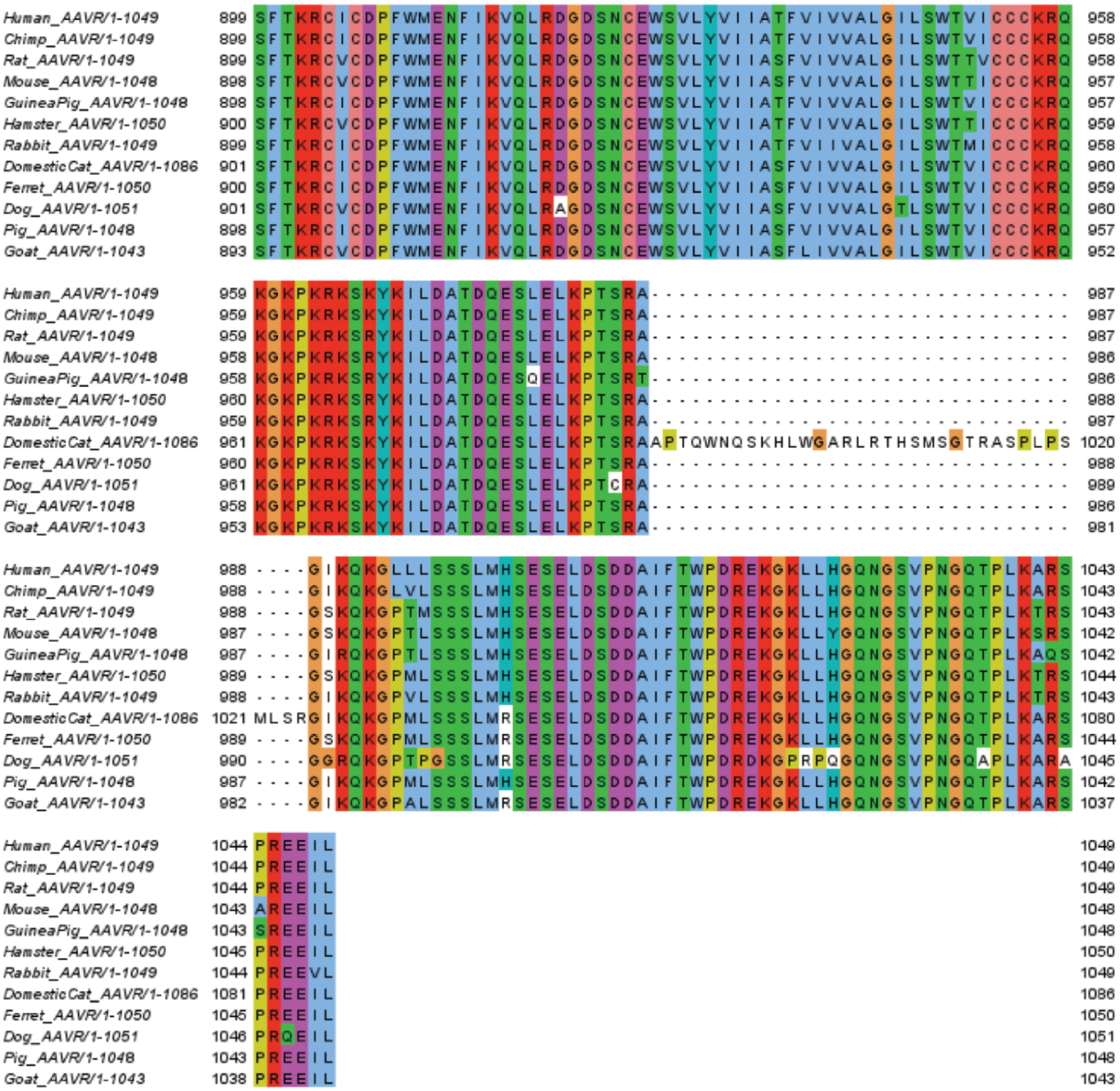

Supplementary Figure S3. Full alignment of AAVR proteins described in Figure 4. Boxed alignments are PKD1-5. Sequences are colored using the Clustal scheme in Jalview.

Experiment	Pair	A_label	B_label	Kd_A_uM	Kd_B_uM	Fold_Kd_B_over_A
BSA only	catPKD1_E353R vs hPKD1_wt	catpkd1_e353r (BSA only)	hpkd1_wt (BSA only)	0.088198077	49.801904	564.6597514
BSA only	catPKD1_wt vs catPKD1_E353R	catpkd1_wt (BSA only)	catpkd1_e353r (BSA only)	0.254143665	0.088198077	0.347040232
BSA only	catPKD1_wt vs hPKD1_R353E	catpkd1_wt (BSA only)	hpkd1_r353e (BSA only)	0.254143665	1.840596295	7.242345758
BSA only	hPKD1_wt vs hPKD1_R353E	hpkd1_wt (BSA only)	hpkd1_r353e (BSA only)	49.801904	1.840596295	0.036958352
Experiment 1	catPKD1_E353R vs hPKD1_wt	catpkd1_e353r	hpkd1_wt	4.645457024	0.328214365	0.070652761
Experiment 1	catPKD1_wt vs catPKD1_E353R	catpkd1_wt	catpkd1_e353r	0.561973344	4.645457024	8.266329846
Experiment 1	catPKD1_wt vs hPKD1_R353E	catpkd1_wt	hpkd1_r353e	0.561973344	1.609847916	2.864633945
Experiment 1	hPKD1_wt vs hPKD1_R353E	hpkd1_wt	hpkd1_r353e	0.328214365	1.609847916	4.904867328
Experiment 2	catPKD1_E353R vs hPKD1_wt	catpkd1_e353r	hpkd1_wt	3.282869183	0.380167371	0.115803387
Experiment 2	catPKD1_wt vs catPKD1_E353R	catpkd1_wt	catpkd1_e353r	0.389767789	3.282869183	8.422628229
Experiment 2	catPKD1_wt vs hPKD1_R353E	catpkd1_wt	hpkd1_r353e	0.389767789	0.886714708	2.27498201
Experiment 2	hPKD1_wt vs hPKD1_R353E	hpkd1_wt	hpkd1_r353e	0.380167371	0.886714708	2.332432438
**Experiment**	**Bmax_A**	**Bmax_B**	**Fold_Bmax_B_over_A**	**Slope_A**	**Slope_B**	**Fold_Slope_B_over_A**
BSA only	0.067455349	3.755783708	55.67807061	0.764816559	0.07541446	0.098604638
BSA only	0.0963411	0.067455349	0.700172092	0.379081256	0.764816559	2.017553087
BSA only	0.0963411	0.386145244	4.008105005	0.379081256	0.209793557	0.553426354
BSA only	3.755783708	0.386145244	0.102813494	0.07541446	0.209793557	2.781874414
Experiment 1	1.862070205	1.759273098	0.944794183	0.400836817	5.360134365	13.37236038
Experiment 1	0.176093455	1.862070205	10.57432945	0.313348412	0.400836817	1.279204876
Experiment 1	0.176093455	0.586417695	3.330150435	0.313348412	0.364269003	1.162504704
Experiment 1	1.759273098	0.586417695	0.333329542	5.360134365	0.364269003	0.067958931
Experiment 2	1.803455718	1.501472055	0.832552771	0.549353513	3.94950269	7.189364585
Experiment 2	0.216205161	1.803455718	8.34140918	0.554702485	0.549353513	0.990357042
Experiment 2	0.216205161	0.621877263	2.876329404	0.554702485	0.701327335	1.264330615
Experiment 2	1.501472055	0.621877263	0.41417838	3.94950269	0.701327335	0.177573581

Supplementary Table S1. This table reports fitted dissociation constants (K_d), maximal ELISA signals (B_{max}), curve slopes, and the corresponding fold-differences for pairwise comparisons of hPKD1_wt, hPKD1_R353E, catPKD1_wt, and catPKD1_E353R across independent ELISA experiments.
